# A Meta-Analysis Shows That Screen Bottom Boards Can Significantly Reduce *Varroa destructor* Population

**DOI:** 10.3390/insects11090624

**Published:** 2020-09-11

**Authors:** Fang Liu, Xinjian Xu, Yuan Zhang, Hongxia Zhao, Zachary Y. Huang

**Affiliations:** 1 Guangdong Key Laboratory of Animal Conservation and Resource Utilization, Guangdong Public Laboratory of Wild Animal Conservation and Utilization, Institute of Zoology, Guangdong Academy of Sciences, Guangzhou 510260, China; lfxiaomifeng@ahau.edu.cn (F.L.); hxzh110@giabr.gd.cn (H.Z.); 2 Department of Entomology, Michigan State University, East Lansing, MI 48824, USA; 3 College of Animal Science (College of Bee Science), Fujian Agriculture and Forestry University, Fuzhou 350002, China; xuxinjian1982@126.com; 4 Yunnan Academy of Biodiversity, Southwest Forestry University, Kunming 650224, China; zygogo@163.com

**Keywords:** screen bottom board, wooden floor, *Varroa destructor*, honeybee

## Abstract

**Simple Summary:**

*Varroa destructor* is the most serious threat to the western honey bee, *Apis mellifera*. Screen bottom board, a cultural method for mite control, is a modified bottom board with a screen to allow mites to fall to a sticky board or to the grass or soil directly below the screen. Most studies show a trend of lower varroa population in colonies with these boards, but the results are usually not statistically significant. To understand whether the negative results were due to small sample sizes, or because the board is actually ineffective, we conducted a meta-analysis with seven published studies with 145 colonies. The results showed that the varroa population in colonies with screen bottom boards is significantly lower compared to those with traditional, wooden floors. The screen bottom board does have a significantly negative impact on the varroa population and can be part of tool kits for mite control.

**Abstract:**

*Varroa destructor* is by far the most serious threat to the western honey bee, *Apis mellifera*. A screen bottom board, a cultural method for mite control, is a modified bottom board with a screen that allows mites to fall onto a sticky board, or the grass or soil below the screen. Whether or not a screen bottom board can reduce varroa significantly has been controversial. Most studies show a trend of lower varroa populations in colonies with these boards, but the results are usually not statistically significant. To understand whether the negative results have been due to small sample sizes, or because the board is actually ineffective, we conducted a meta-analysis with seven published studies with a total of 145 colonies. Meta-analysis showed that the confidence intervals of the combined effect sizes were negative with a Hedges’ g of −1.09 (SE 0.39, 95% CI −2.0 to −0.19, *p* < 0.01), which suggests that the varroa population in colonies with screen bottom boards is significantly lower compared to those with traditional wooden floors. We thus conclude that the screen bottom board does have a significantly negative impact on the varroa population and can be part of tool kits for mite control.

## 1. Introduction

Honey bees (*Apis spp.*) are important agricultural pollinators, especially in the United States. Over $14 billion is attributed to honey bee pollination in US agriculture [1]. One third of the food we eat directly or indirectly is pollinated by honey bees [2]. However, the health of honey bees has been declining. For example, in the United States, the average yearly mortality of honey bee colonies has exceeded 50% [3]. Many factors have been blamed for this high mortality, including the varroa mite (*Varroa destructor*), pathogens, pesticide use, loss of habitat and transportation. *Varroa destructor*, which was originally associated with the Asian honey bee, *Apis cerana*, now mainly targets the European honey bee, *Apis mellifera*, which has little resistance to it. Since the 1960s, varroa has spread from Asia to Europe, the Americas, to New Zealand and now nearly the whole world [4].

*Varroa destructor* feeds on the hemolymph/fat body of the honey bee [5,6]. It also transmits many honey bee viruses and diseases [7]. There are many methods used in controlling mites, the most popular of which is to use chemicals, which include hard and soft acaricides [8]. Some of these are effective in controlling mites, but treatments can become less effective due to the development of resistance and can also leave residue in honey [4]. Comparatively, nonchemical controls are safer to the bees and environment. There are several nonchemical methods to control mites, including drone removal, heat treatments, powdered sugar and screen bottom boards [8]. Among these, the screen bottom board is the simplest method and is used widely. When varroa mites fall off bees by accident, or are removed by bees due to their grooming behavior [9], they fall down to the bottom of the hive where they have a chance to reinfest the colony. The screen bottom boards can slow the increase of varroa mites, as they separate fallen mites and bees, preventing mites from returning [10,11]. A standard bottom board of a Langstroth-style hive contains a solid wooden floor; when mites fall onto the floor, 40–50% of them live and are given a second chance at the honey bees [12]. Screen bottom boards were designed by Pettis and Shimanuki [10]. They designed two types of screen bottom boards, one with a wire mesh screen with a sticky white paper below it (sticky bottom board), and the other a mesh bottom board (also named open mesh floors or open screen floor) with no solid board below the screen. Numerous studies have tested the effectiveness of these devices, which both feature a floor comprised of 8-mesh hardware cloth (3.15 squares per cm) [13,14,15,16,17,18,19]. However, the majority of these studies did not detect statistical differences in mite reduction. These results could be due to two very different reasons: 1. that there was no true effect of this method in reducing mite population, or 2. that the sample sizes used in the studies were too small.

In this study, data from seven studies on the efficacy of screen bottom boards (including mesh floors, closed screen bottom boards and open screen bottom boards) over wooden floors in controlling mites was analyzed by meta-analysis [20]. The results showed that varroa mite density in colonies with screen bottom boards was significantly lower than those with wooden floors.

## 2. Materials and Methods

### 2.1. Collection of Data

Studies about the efficacy of controlling mites using bottom screen boards were collected through several searching tools, including Google Scholar, NCBI and ISI Web of Science. The keywords for searching were honey bee, varroa mite, screen bottom board or wooden floors. A total of 25 references were found, and seven of them were chosen in our study according to the following criteria: 1. references should study mites in honey bees and not other insects; 2. the data should contain either mean numbers of natural mite fall (NMF) or mite density (MD) and with errors (standard errors or standard deviations); 3. studies where the screen bottom board was tested in combination with pesticides were excluded; and 4. studies with no available data or duplicated (studies reporting results already included in another publication) were excluded.

### 2.2. Digitalization of Data from Figures

Three out of seven studies [10,17,19] showed their results with only figures. We captured the figures in pdf files, then used WebPlotDigitizer (version 3.8) to convert the figures to the means and standard errors for further analysis [21]. For example, we captured two points (a and b) on the first column of Figure 1 from Coffey (2007) [17]. The value of ‘a’ was identified as the mean of natural mite fall of samples with a normal floor, and the value of ‘b–a’ (b minus a) was identified as the corresponding standard error (Figure S1). Both of the values were used for future analysis. The values from the other two figures were obtained using the same method. All data captured are provided in the Supplementary Materials (Tables S1–S3).

### 2.3. Relationship between Natural Mite Fall and Mite Density in Colonies

In published studies on the use of screen bottom boards, some reported MD, while others used NMF per 24 h, 48 h or 72 h. We, therefore, needed to establish a relationship between the two parameters so that NMF could be converted into MD, which was used in our analysis. Thirteen colonies of similar strength were used in the present study, containing two boxes (one deep and one medium) each with about 12–14 frames of bees. Worker bees (*n* = 300) from each colony were shaken with powdered sugar to dislodge mites [22] and the numbers of mites (Mi) were then counted. The mite density (MD) of bees from each colony was calculated as MD = Mi/300 [23]. At the same time, we measured the natural mite fall (NMF) of these colonies by inserting a piece of plastic board (B92101, Dadant.com) with vegetable oil sprayed on the upper surface in October of 2018 in the apiary at Michigan State University. The board was retrieved after 48 h and NMF was counted and adjusted as NMF per 24 h. We then established a relationship between MD and NMF using linear regression analysis (StatView 5.0.1). Finally, using this relationship we converted published NMF data into MD for further analysis (see 2.4 below).

### 2.4. Data Conversion

MD in colonies was used for final analysis in our study. We obtained MD in colonies from each reference. Four of the seven studies chosen [10,16,17,18] showed their results with natural mite fall. We converted these numbers into MD in colonies according to the relationship obtained in our own study (see Section 2.3). There were two data sets from two independent experiments in the study of Harbo and Harris [15], one from the 20th day of the experiment, the other from the 68th day of the experiment. These were combined for further analysis. Two other data were from the study of Sammataro et al., as they did two independent experiments with colonies from two different sites [16].

The MD from the other two studies [13,19] was calculated directly using the number of mites divided by the total number of honey bees in each colony, because they had the exact population of bees also.

Four studies [10,17,18,19] had multiple datasets (sampled at different times). We combined these data into one data point per group (treatment or control) by averaging all data points. Data from each group ($\bar{x}$ average; SD: standard deviation) (Table S4) was normalized first (divided by $\bar{x}\;$ of wooden floor, then divided by 10), then an average was calculated for meta-analysis. The results of converted data are shown in Table 1.

### 2.5. Data Analysis

Hedges’ g was calculated for each study as the difference between the average ($\bar{x}$) of screen bottom boards and wooden floors divided by the pooled standard deviation (SD) and weighted by the reciprocal of the sampling variance [20]. The sign of Hedges’ g was reversed for mite density (MD). Therefore, negative values would indicate lower MD in colonies with a screen bottom board, while a positive value indicates higher MD in colonies with a screen bottom board. A 95% confidence interval (CI) was used to determine if specific effect size of a study differed significantly from zero. Forest plots were made for all outcomes displaying the effective size of each study and 95% confidence interval. If the confidence interval of the combined effect size does not include zero, in the case of a confidence level of 95%, then the *p*-value is smaller than 0.05. It means that the meta-analytic effect is statistically significant.

## 3. Results

**Figure 1:**
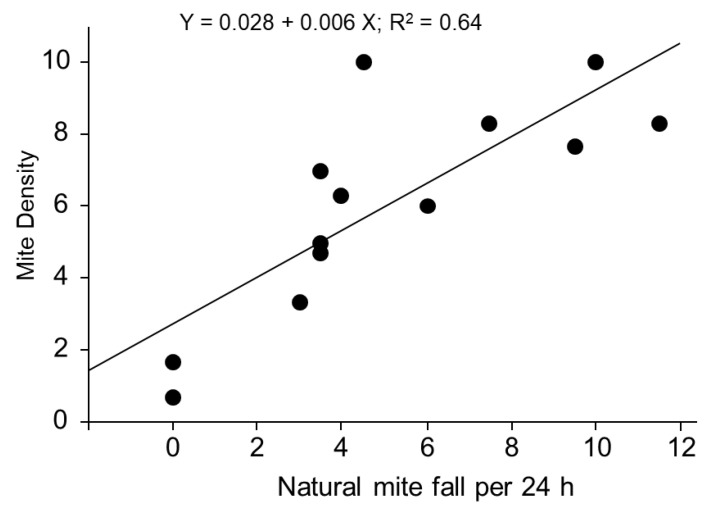
There was a significant correlation (regression analysis, *p* < 0.01) between natural mite fall and mite density.

As most of the references we used calculated the NMF of colonies, we established a relationship between NMF and MD in order to standardize our analysis using only MD. With the NMF and MD of 13 colonies, we obtained a significant positive relationship between the two parameters (Figure 1). The equation was Y = 0.028 + 0.006 * X, R^2^ = 0.636, where Y represents the MD in a colony and X representing the number of NMF during a 24 h period.

**Figure 2:**
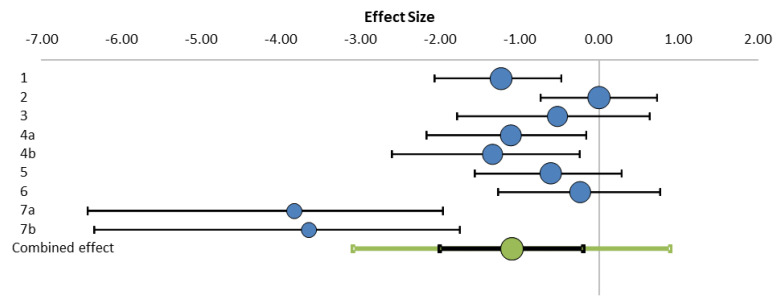
Forest plot of mite density in colonies (screen bottom board vs. wooden floor) from seven studies.

The *x*-axis of the Figure 2 forms the effect size scale, plotted on the top of the plot. Each row, except the bottom one, represents a study’s effect size estimate in the form of a point and a (95%) CI. The point estimate is represented in the forest plot by a smaller or a larger bullet. The bottom row of the forest plot represents the result of meta-analysis. It consists of two intervals around the same bullet, which represents the weight average effect. The smaller, black interval is a confidence interval, whereas the bigger, green interval is the prediction interval. The relative size of these bullets represents each study’s weight in the generation of meta-analysis. We can see the effect sizes of all seven studies. Some studies (No. 1, 4, 5, 7) with effects have shown statistically significant negative effects, other studies (No. 2, 3, 6) show effects that are statistically nonsignificant (Table 1). However, the value of the combined effect size is −1.09 and its confidence interval is 95% CI −2.0 to −0.19, which does not include zero. Furthermore, the confidence level of 95% has a *p*-value smaller than 0.01. The results suggest that the overall effect of screen bottom boards in reducing mite population is significant.

## 4. Discussion

Numerous studies have focused on calculating the numbers of mites for an evaluation of the mite density in colonies. Some of them [24,25,26] evaluate level of mites by counting the dead mites on sticky-boards after treatment with acaricide. Others sample the natural mortality of mites to evaluate mite infestation [27]. None of these were suitable for the present study. Most of the seven studies we analyzed used NMF, so we established a relationship between NMF and MD in colonies to better analyze the limited number of studies. Fries and his colleagues (1991) found a similar relationship (r^2^ = 0.65) between daily mite downfall and mites per live bee [28] with a larger sample size (35 colonies). Unfortunately, no mathematical relationship (regression line) was given, so we could not use their data for our study. Similarly, Branco et al. (2006) found a very close relationship (r^2^ = 0.84) between the weekly dead mites (similar to our natural mite fall) and total mite population estimate [29]. Unfortunately, no relationship was given between weekly dead mites and percentage of infestation on adult bees. It appears that they had the data, but they did not present them in the paper (nor in Supplementary Materials). In this study, we established a relationship between the natural mite fall and MD in 13 colonies by using the sugar dusting method, which helped us convert the natural mite fall into mites per bee for further study. Colony strength is a very important factor for mite fall because larger colonies have more mites even with the same mite density as smaller colonies. All the references we used started their experiments with strong colonies with adult bees of 10,000~25,000. In this study, we used 13 colonies of similar strengths. Each colony contained two boxes (one deep and one medium) and each had about 12–14 deep frames of bees (~40,000–49,000 adult bees). Our colonies were therefore 2–5 times stronger than the cited studies and there might be errors associated with estimating mite numbers due to this. Because most of the data we used were from the United States, and there is a possibility that different subspecies of bees might affect natural mite fall, we tried to establish this relationship for ourselves using U.S. honey bees.

We found that the confidence intervals of the combined effect sizes were negative with a Hedges’ g of −1.09 (SE 0.39, 95% CI −2.0 to −0.19, *p* < 0.01), suggesting that the varroa population in colonies with screen bottom boards is significantly lower than those with traditional wooden floors. Researchers have tested the effects of screen floors on overwintering [15,30], honey consumption [15] and brood production [13,15,31]. However, the direct effect of screen floors on controlling mites is unclear. In our meta-analysis, we can see that some confidence intervals of studies were entirely negative, while other confidence intervals included zero (Figure 2). However, the confidence interval of the combined effect size was entirely negative, which means that the screen bottom board effect is statistically significant.

Findings from our meta-analysis suggest that screen bottom boards can significantly reduce mite populations compared to wooden floors. Some studies showed that the screen bottom boards are effective in controlling mites when they are combined with other methods. Ashar et al. demonstrated that screen bottom boards had a significant effect on controlling mites when combined with a powdered sugar treatment [32]. Mahmood et al. reported that screen bottom boards alone can effectively control varroa mite populations, and they showed significantly higher efficacy when they were used together with soft chemicals, and without any side effects [33]. Deplaplane et al. showed that the screen bottom board had an effect in reducing colony varroa mite levels, though it proved more effective when the colonies had hygienic queens [18]. Though we have confirmed that screen bottom boards as a nonchemical control measure was a very useful tool in beekeeping management, the number of studies we included here was quite limited. Further studies should be performed to verify the best conditions for using screen bottom boards in managing varroa mites.

## 5. Conclusions

In this study, a meta-analysis was conducted with seven published studies to understand whether screen bottom board does have a significantly negative impact on the varroa population. The results showed that the confidence intervals of the combined effect sizes were negative with a Hedges’ g of −1.09 (SE 0.39, 95% CI −2.0 to −0.19, *p* < 0.01), which suggests that the varroa population in colonies with screen bottom boards is significantly lower compared to those with traditional wooden floors. We thus conclude that the screen bottom boards can significantly reduce *Varroa destructor* population and can be part of tool kits for mite control. 

## Figures and Tables

**Table 1:** Mite density of colonies settled with wooden floor and screen bottom board from seven studies.

No.	Study	Wooden Floor			Screen Bottom Board		
		$\bar{x}$	SD	N	$\bar{x}$	SD	N
1	Coffey (2007) [17]	0.1	0.0604	15	0.0809	0.0080	15
2	Delaplane et al. (2005) [18]	0.1	0.0799	14	0.0961	0.0981	16
3	Ellis et al. (2001) [13]	0.0996	0.0661	6	0.0838	0.0857	6
4a	Harbo and Harris (2004) [15]	0.1	0.0514	9	0.0989	0.0312	10
4b	Harbo and Harris (2004) [15]	0.1	0.0541	7	0.0788	0.0503	8
5	Pettis and Shimanuki (1999) [10]	0.1	0.0524	10	0.0854	0.0524	10
6	Rinderer et al. (2003) [19]	0.1	0.1205	8	0.0875	0.1205	8
7a	Sammataro et al. (2004) [16]	0.144	0.0402	5	0.0826	0.0441	6
7b	Sammataro et al. (2004) [16]	0.2676	0.1632	5	0.0552	0.1632	5

$\bar{x}\;$ represents the mean of mite density, SD represents the standard deviation, N represents the number of colonies.

## Supplementary Materials

The following are available online at https://www.mdpi.com/2075-4450/11/9/624/s1, Table S1: The data captured from Figure 2 of Pettis and Shimanuki (1999), Table S2: The data captured from Figure 1 of Coffey (2007), Table S3: The data captured from Figure 2 of Rinderer et al. (2003), Table S4: Mite infestation rates of colonies with screen bottom boards and wooden floor from seven studies. They were then normalized (each divided by $\bar{x}$ of wooden floor and then 10, and averaged, to produce a single data print for each treatment, which is presented in Table 1), Figure S1: Explaining of capturing data from Figures (take Figure 1 of Coffey, 2007 for example). Two points (“a” and “b”) on the first column of Figure 1 were captured by WebPlotDigitizer (version 3.8) from Coffey (2007). The value of “a” was identified as the mean of natural mite fall of samples with normal floor, and the value of “b-a” (b subtracts a) was identified as the corresponding error.
