# Supplementary material for: A Meta-Analysis Shows That Screen Bottom Boards Can Significantly Reduce Varroa destructor Population
Source: Insects. 2020 Sep 11;11(9):624. doi: 10.3390/insects11090624 (PMC7564001; doi:10.3390/insects11090624)
Supplement: Supplementary file 1 [file insects-11-00624-s001.zip › supplementary materials/Figure S1.pptx]

## Slide 1
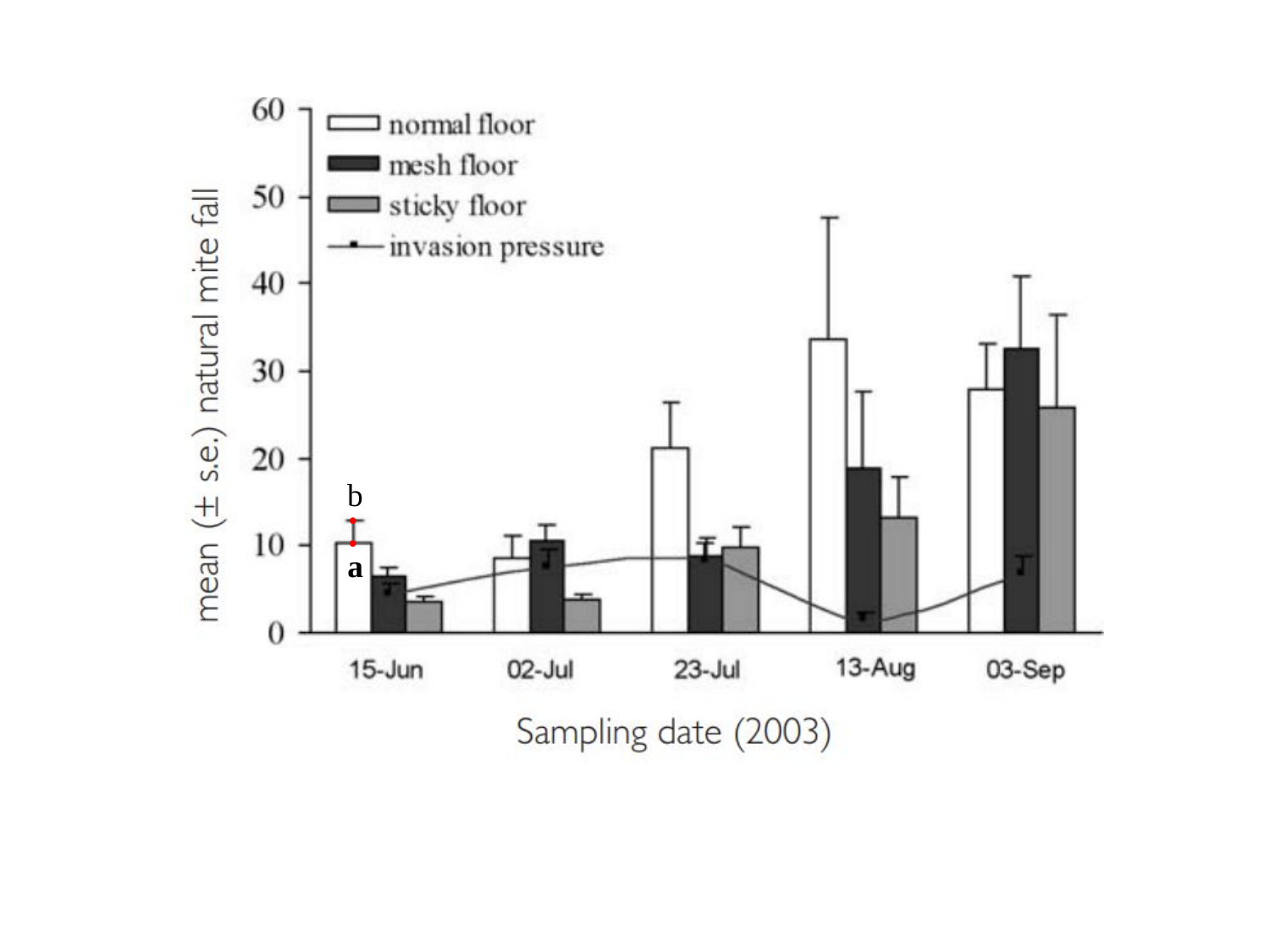

b
●
●
a

## Slide 2
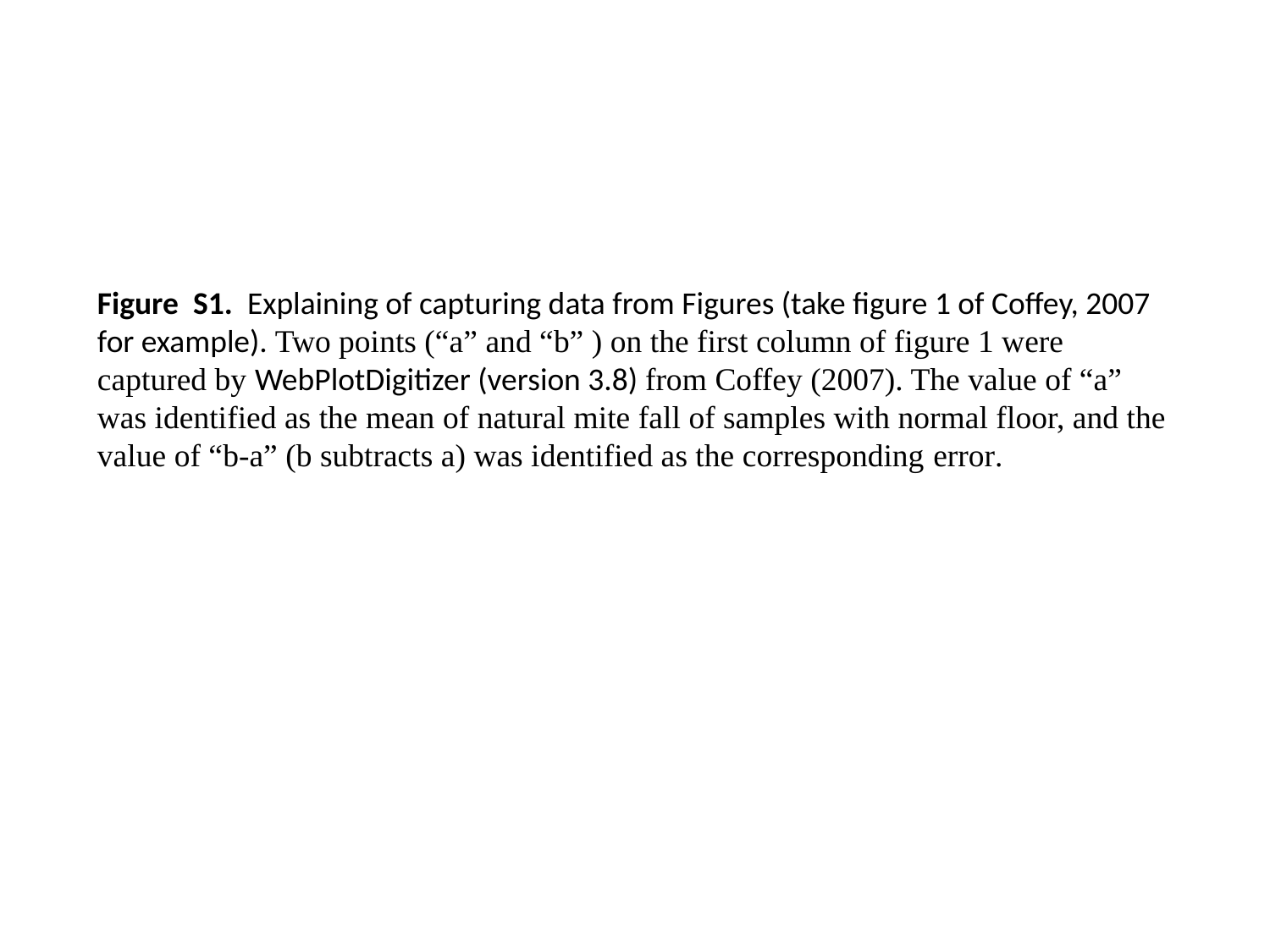

Figure S1. Explaining of capturing data from Figures (take figure 1 of Coffey, 2007 for example). Two points (“a” and “b” ) on the first column of figure 1 were captured by WebPlotDigitizer (version 3.8) from Coffey (2007). The value of “a” was identified as the mean of natural mite fall of samples with normal floor, and the value of “b-a” (b subtracts a) was identified as the corresponding error.
