# Supplementary material for: A Meta-Analysis Shows That Screen Bottom Boards Can Significantly Reduce Varroa destructor Population
Source: Insects. 2020 Sep 11;11(9):624. doi: 10.3390/insects11090624 (PMC7564001; doi:10.3390/insects11090624)
Supplement: Supplementary file 1 [file insects-11-00624-s001.zip › supplementary materials/Table S1.docx]

**Table S1.** The data captured from Figure 2 of Pettis and Shimanuki (1999).

| Wood floor | | | Sticky floor | | | Mesh floor | | | Date |
| --- | --- | --- | --- | --- | --- | --- | --- | --- | --- |
| M | SE | N | M | SE | N | M | SE | N |  |
| 39.36 | 5.95 | 10 | 35.72 | 4.78 | 10 | 37.52 | 7.10 | 10 | June |
| 80.21 | 12.86 | 10 | 68.51 | 10.55 | 10 | 68.00 | 9.43 | 10 | July |
| 263.96 | 69.32 | 10 | 191.20 | 27.84 | 10 | 191.87 | 39.36 | 10 | Aug |

M represents the mean of number of fallen varroa, SE represents the standard error, N represents the number of colonies,

SE would be transformed into SD for meta-analysis.
