# Supplementary material for: A Meta-Analysis Shows That Screen Bottom Boards Can Significantly Reduce Varroa destructor Population
Source: Insects. 2020 Sep 11;11(9):624. doi: 10.3390/insects11090624 (PMC7564001; doi:10.3390/insects11090624)
Supplement: Supplementary file 1 [file insects-11-00624-s001.zip › supplementary materials/Table S2.docx]

**Table S2.** The data captured from Figure 1 of Coffey (2007).

| Wood floor | | | Sticky floor | | | Mesh floor | | | Date |
| --- | --- | --- | --- | --- | --- | --- | --- | --- | --- |
| M | SE | N | M | SE | N | M | SE | N |  |
| 10.33 | 2.55 | 15 | 3.40 | 0.57 | 15 | 6.65 | 0.71 | 15 | 15-Jun |
| 8.49 | 2.41 | 15 | 3.68 | 0.71 | 15 | 10.61 | 1.70 | 15 | 2-Jul |
| 21.08 | 5.38 | 15 | 9.76 | 2.41 | 15 | 8.77 | 2.12 | 15 | 23-Jul |
| 33.68 | 14.01 | 15 | 13.16 | 4.67 | 15 | 18.96 | 8.77 | 15 | 13-Aug |
| 28.02 | 5.09 | 15 | 25.90 | 10.61 | 15 | 32.83 | 8.07 | 15 | 03-Step |

M represents the mean of natural mite fall, SE represents the standard error, N represents the number of colonies.

SE would be transformed into SD for meta-analysis.
