# Supplementary material for: A Meta-Analysis Shows That Screen Bottom Boards Can Significantly Reduce Varroa destructor Population
Source: Insects. 2020 Sep 11;11(9):624. doi: 10.3390/insects11090624 (PMC7564001; doi:10.3390/insects11090624)
Supplement: Supplementary file 1 [file insects-11-00624-s001.zip › supplementary materials/Table S4.docx]

**Table S4.** Mite infestation rates of colonies with screen bottom boards and wooden floor from seven studies. They were then normalized (each divided by $\bar{x}$ of wooden floor and then 10, and averaged, to produce a single data print for each treatment, which is presented in Table 1).

| Studies | Wooden floor | | | Screen bottom board | | |
| --- | --- | --- | --- | --- | --- | --- |
|  | $\bar{x}$ | SD | N | $\bar{x}$ | SD | N |
| Coffey (2007) | 0.0487 | 0.0198 | 15 | 0.0348 | 0.0025 | 15 |
| Coffey (2007) | 0.0450 | 0.0186 | 15 | 0.0354 | 0.0025 | 15 |
| Coffey (2007) | 0.0702 | 0.0418 | 15 | 0.0475 | 0.0049 | 15 |
| Coffey (2007) | 0.0954 | 0.1084 | 15 | 0.0543 | 0.0091 | 15 |
| Coffey (2007) | 0.0840 | 0.0395 | 15 | 0.0798 | 0.0081 | 15 |
| Coffey (2007) | 0.0487 | 0.0198 | 15 | 0.0413 | 0.0029 | 15 |
| Coffey (2007) | 0.0450 | 0.0186 | 15 | 0.0492 | 0.0034 | 15 |
| Coffey (2007) | 0.0702 | 0.0418 | 15 | 0.0455 | 0.0047 | 15 |
| Coffey (2007) | 0.0954 | 0.1084 | 15 | 0.0659 | 0.0110 | 15 |
| Coffey (2007) | 0.0840 | 0.0395 | 15 | 0.0937 | 0.0095 | 15 |
| Delaplane et al. (2005) | 0.0340 | 0.0080 | 20 | 0.0322 | 0.0078 | 19 |
| Delaplane et al. (2005) | 0.0400 | 0.0107 | 20 | 0.0412 | 0.0107 | 20 |
| Delaplane et al. (2005) | 0.0652 | 0.0445 | 19 | 0.0598 | 0.0456 | 20 |
| Delaplane et al. (2005) | 0.1006 | 0.0840 | 18 | 0.0886 | 0.0885 | 20 |
| Delaplane et al. (2005) | 0.0946 | 0.0764 | 18 | 0.0712 | 0.0805 | 20 |
| Delaplane et al. (2005) | 0.0694 | 0.0732 | 19 | 0.0448 | 0.0751 | 20 |
| Delaplane et al. (2005) | 0.0652 | 0.0303 | 13 | 0.0814 | 0.0346 | 17 |
| Delaplane et al. (2005) | 0.1084 | 0.0909 | 13 | 0.0934 | 0.0976 | 15 |
| Delaplane et al. (2005) | 0.1708 | 0.1579 | 13 | 0.1606 | 0.1752 | 16 |
| Delaplane et al. (2005) | 0.2350 | 0.3483 | 13 | 0.0886 | 0.3864 | 16 |
| Delaplane et al. (2005) | 0.1030 | 0.0806 | 2 | 0.2236 | 0.1612 | 8 |
| Delaplane et al (2005) | 0.3130 | 0.3776 | 2 | 0.2368 | 0.6540 | 6 |
| Ellis et al. (2001) | 0.0996 | 0.0661 | 6 | 0.0838 | 0.0857 | 6 |
| Harbo and Harris (2004) | 0.0043 | 0.0024 | 9 | 0.0053 | 0.0015 | 10 |
| Harbo and Harris (2004) | 0.0108 | 0.0051 | 9 | 0.0081 | 0.0030 | 10 |
| Harbo and Harris (2004) | 0.0078 | 0.0035 | 7 | 0.0113 | 0.0043 | 7 |
| Harbo and Harris (2004) | 0.0078 | 0.0035 | 7 | 0.0060 | 0.0045 | 7 |
| Harbo and Harris (2004) | 0.0333 | 0.0213 | 7 | 0.0178 | 0.0196 | 8 |
| Harbo and Harris (2004) | 0.0333 | 0.0213 | 7 | 0.0139 | 0.0098 | 8 |
| Pettis and Shimanuki (1999) | 0.1067 | 0.0376 | 10 | 0.0994 | 0.0376 | 10 |
| Pettis and Shimanuki (1999) | 0.1884 | 0.0813 | 10 | 0.1650 | 0.0813 | 10 |
| Pettis and Shimanuki (1999) | 0.5559 | 0.4383 | 10 | 0.4104 | 0.4383 | 10 |
| Pettis and Shimanuki (1999) | 0.1067 | 0.0376 | 10 | 0.1030 | 0.0376 | 10 |
| Pettis and Shimanuki (1999) | 0.1884 | 0.0813 | 10 | 0.1640 | 0.0813 | 10 |
| Pettis and Shimanuki (1999) | 0.5559 | 0.4383 | 10 | 0.4117 | 0.4383 | 10 |
| Rinderer et al. (2003) | 0.0130 | 0.0277 | 8 | 0.0104 | 0.0277 | 8 |
| Rinderer et al. (2003) | 0.1058 | 0.0294 | 8 | 0.1005 | 0.0294 | 8 |
| Sammataro et al. (2004) | 0.1440 | 0.0402 | 5 | 0.0826 | 0.0441 | 6 |
| Sammataro et al. (2004) | 0.2676 | 0.1632 | 5 | 0.0552 | 0.1632 | 5 |

$\bar{x}$ represents the average of mite density, SD represents the standard deviation, N represents the number of colonies.
