# Supplementary material for: A Meta-Analysis Shows That Screen Bottom Boards Can Significantly Reduce Varroa destructor Population
Source: Insects. 2020 Sep 11;11(9):624. doi: 10.3390/insects11090624 (PMC7564001; doi:10.3390/insects11090624)
Supplement: Supplementary file 1 [file insects-11-00624-s001.zip › supplementary materials/Table S3.doc]

**Table S3.** The data captured from Figure 2 of Rinderer et al. (2003)

| Wood floor | | | Mesh floor | | | Date |
| --- | --- | --- | --- | --- | --- | --- |
| M | SE | N | M | SE | N |  |
| 289.46 | 217.19 | 8 | 230.11 | 211.12 | 8 | Jul |
| 2269.09 | 223.25 | 8 | 2155.54 | 271.36 | 8 | Oct |

M represents the average number of mite, SE represents the standard error, N represents the number of colonies. SE would be transformed into SD for meta-analysis.
